# Supplementary figures and images for: Expression, purification and initial characterization of human serum albumin domain I and its cysteine 34
Source: PLoS One. 2020 Oct 12;15(10):e0240580. doi: 10.1371/journal.pone.0240580 (PMC7549792; doi:10.1371/journal.pone.0240580)

Images were obtained using a Canon scanner

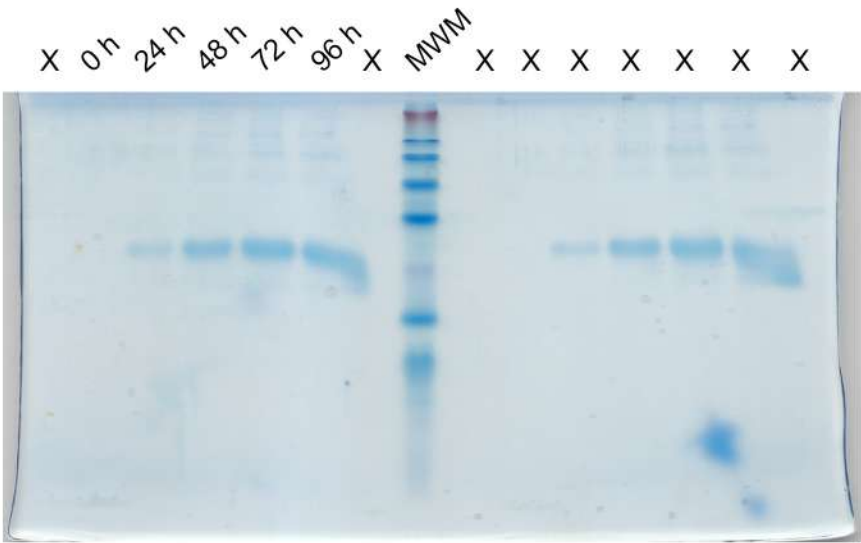

Fig 1 panel A

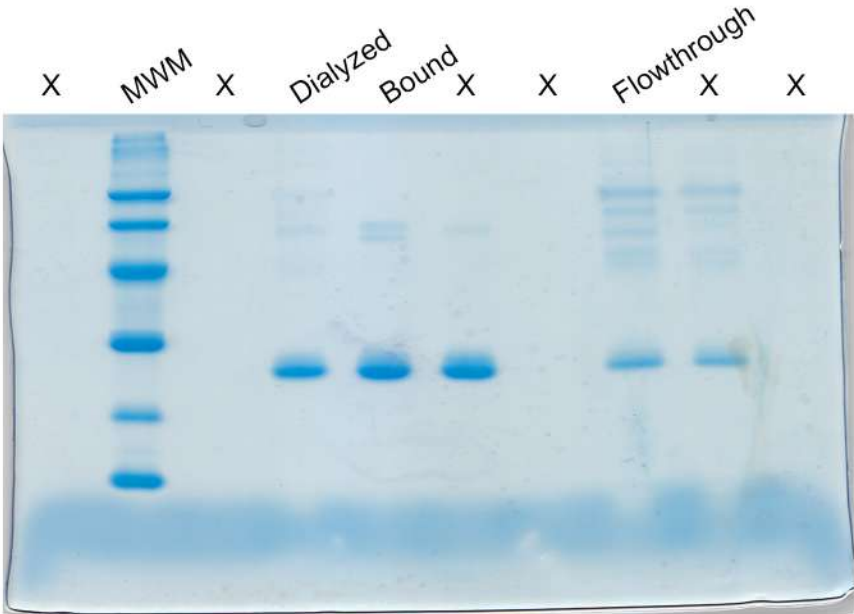

Fig 2 panel B

Supplement: S1 Raw images — (PDF) [file pone.0240580.s001.pdf]
